# Supplementary material for: Exploring Relevant mRNAs and miRNAs in Injured Urethral Tissues of Rats with High-Throughput Sequencing
Source: Genes (Basel). 2022 May 5;13(5):824. doi: 10.3390/genes13050824 (PMC9141346; doi:10.3390/genes13050824)
Supplement: Supplementary file 1 [file genes-13-00824-s001.zip › Supplementary Figure.pdf]

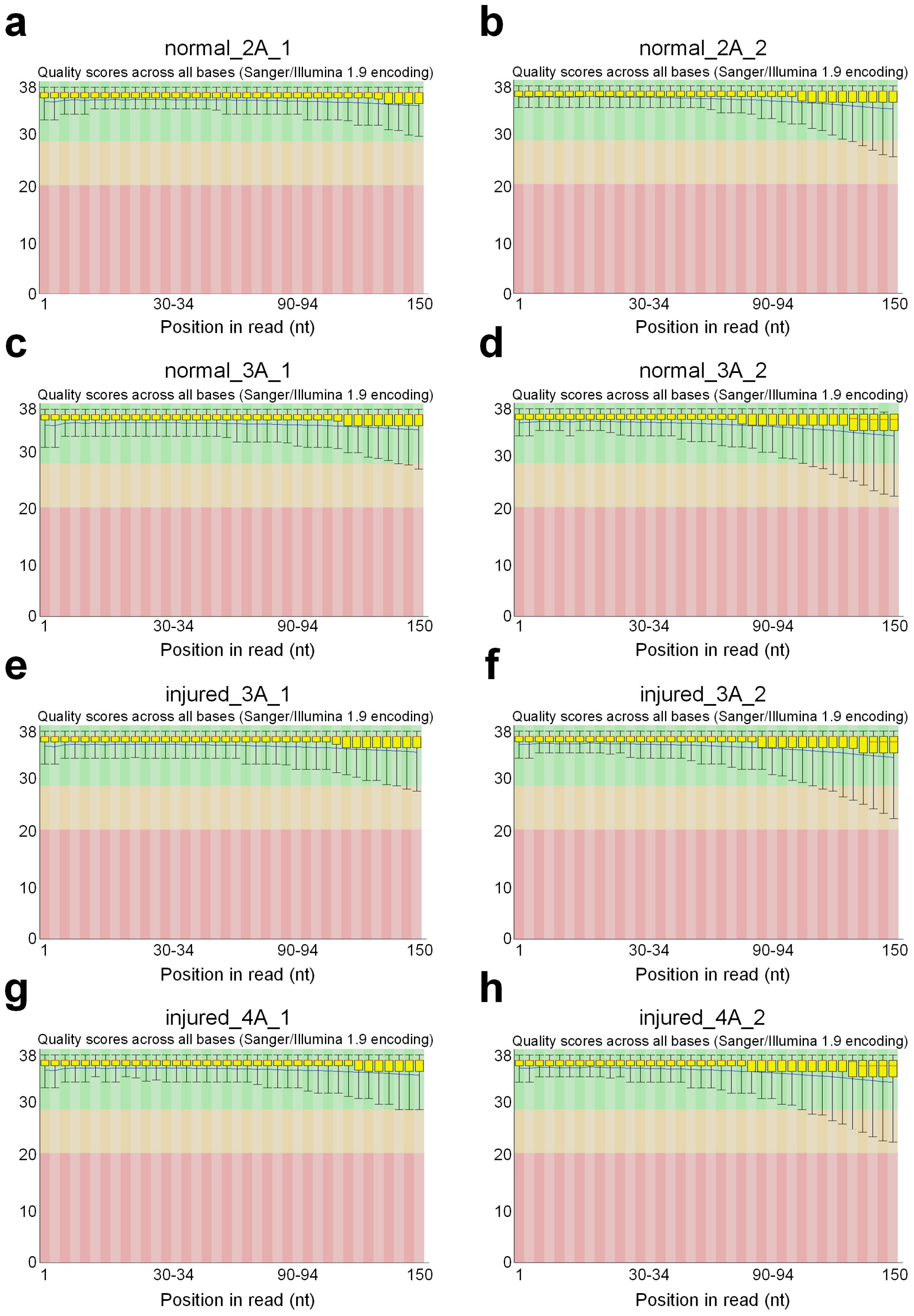

Figure S1. Per base sequence quality of 4 RNA-seq sequencing profiles (**a–h**). The y-axis is quality scores.

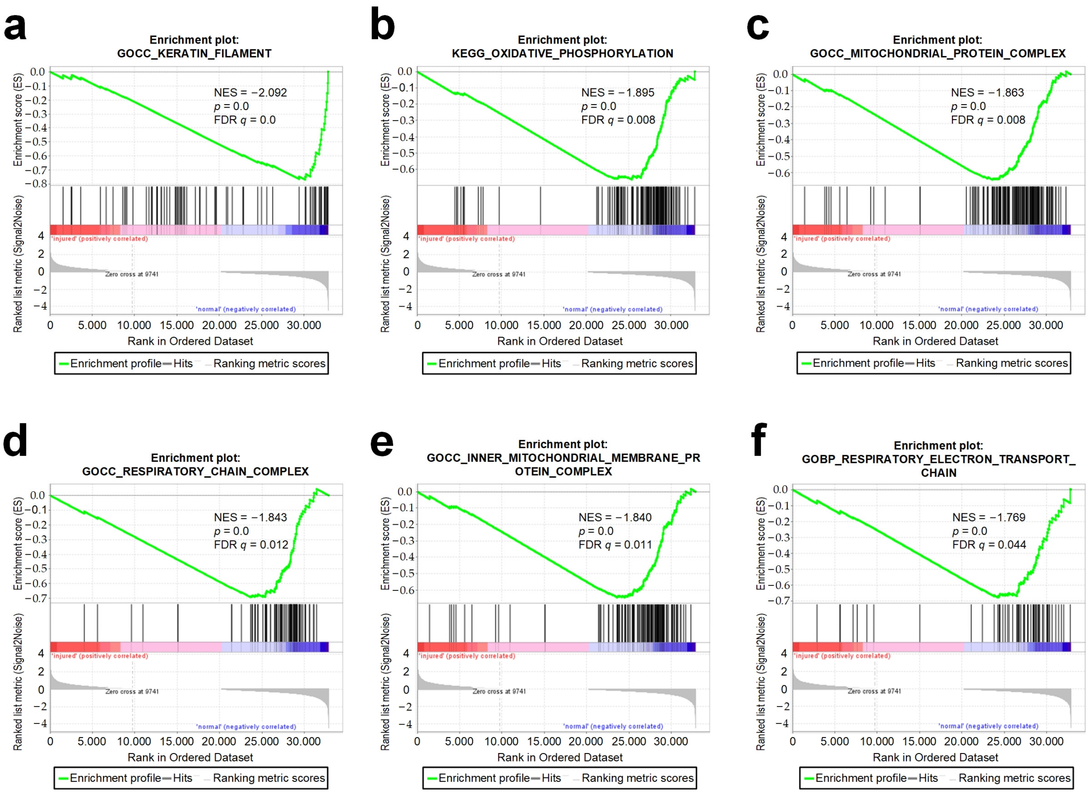

Figure S2. GSEA analysis of 6 representative significantly enriched gene sets in phenotype normal. Enrichment plots comparing normal toward injured phenotype were depicted with following sets of genes: (**a**) GOCC keratin filament, (**b**) KEGG oxidative phosphorylation, (**c**) GOCC mitochondrial protein complex, (**d**) GOCC respiratory chain complex, (**e**) GOCC inner mitochondrial membrane protein complex and (**f**) GOBP respiratory electron transport chain. Comparison of samples, NES, nominal *p*-value, and FDR *q*-value were determined by the GSEA software, and were indicated within each enrichment plot.

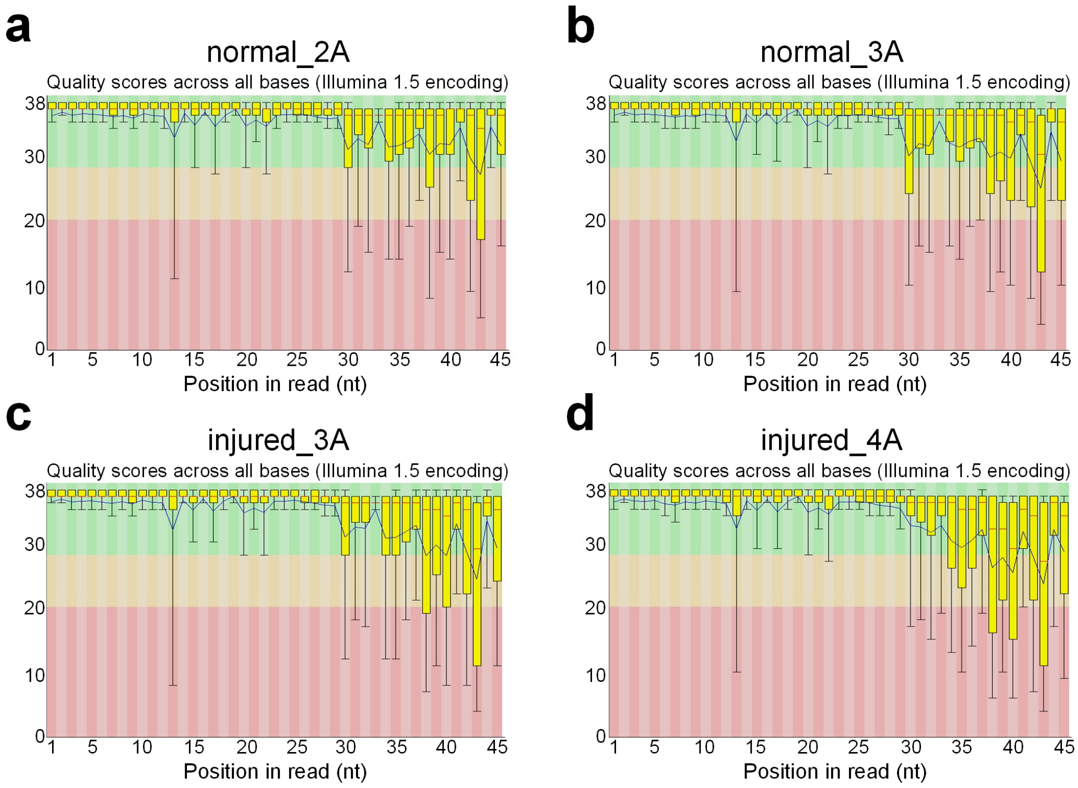

Figure S3. Per base sequence quality of 4 small RNA sequencing profiles (**a–d**). The y-axis is quality scores.

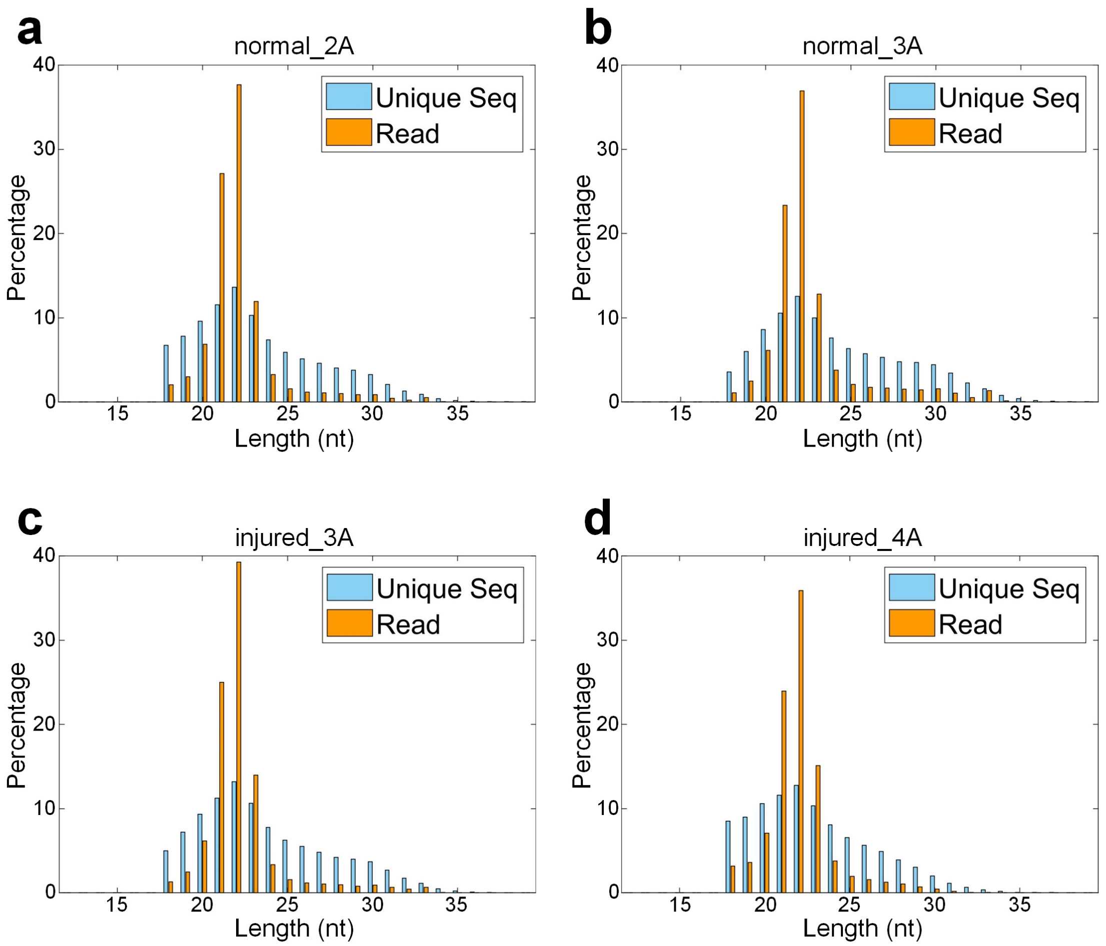

Figure S4. The length distributions of reads and unique sequences of 4 sRNA-seq profiles of rat urethra tissues (**a–d**).
